# High rate of loss to follow-up and virological non-suppression in HIV-infected children on antiretroviral therapy highlights the need to improve quality of care in South Africa

**DOI:** 10.1017/S0950268821000637

**Published:** 2021-03-22

**Authors:** Geneviève A. F. S. van Liere, Rivka Lilian, Jackie Dunlop, Carol Tait, Kate Rees, Moya Mabitsi, Lucy Ranoto, Helen E. Struthers, James A. McIntyre, Remco P. H. Peters

**Affiliations:** 1 Anova Health Institute, Johannesburg, South Africa; 2 Department of Sexual Health, Infectious Diseases and Environmental Health, Public Health Service South Limburg, Heerlen, the Netherlands; 3 Department of Medical Microbiology Maastricht University Medical Centre (MUMC+) and Care and Public Health Research Institute (CAPHRI), Maastricht University, Maastricht, the Netherlands; 4 Division of Community Paediatrics, School of Paediatrics, University of the Witwatersrand, Johannesburg, South Africa; 5 Department of Community Health, School of Public Health, University of the Witwatersrand, Johannesburg, South Africa; 6 Division of Infectious Diseases & HIV Medicine, Department of Medicine, University of Cape Town, Cape Town, South Africa; 7 School of Public health, University of Cape Town, Cape Town, South Africa; 8 Division of Epidemiology and Biostatistics, School of Public Health, Faculty of Health Sciences, University of the Witwatersrand, Johannesburg, South Africa

## Abstract

Provision of high-quality care and ensuring retention of children on antiretroviral therapy (ART) are essential to reduce human immunodeficiency virus (HIV)-associated morbidity and mortality. Virological non-suppression (≥1000 viral copies/ml) is an indication of suboptimal HIV care and support. This retrospective cohort study included ART-naïve children who initiated first-line ART between July 2015 and August 2017 in Johannesburg and rural Mopani district. Of 2739 children started on ART, 29.5% (807/2739) were lost to care at the point of analysis in August 2018. Among retained children, overall virological non-suppression was 30.2% (469/1554). Virological non-suppression was associated with higher loss to care 30.3% (229/755) compared with suppressed children (9.7%, 136/1399, *P* < 0.001). Receiving treatment in Mopani was associated with virological non-suppression in children under 5 years (adjusted odds ratio (aOR) 1.7 (95% confidence interval (CI) 1.1–2.4), 5–9 years (aOR 1.8 (1.1–3.0)) and 10–14 years (aOR 1.9 (1.2–2.8)). Virological non-suppression was associated with lower CD4 count in children 5–9 years (aOR 2.1 (1.1–4.1)) and 10–14 years (aOR 2.1 (1.2–3.8)). Additional factors included a shorter time on ART (<5 years aOR 1.8–3.7 (1.3–8.2)), and male gender (5–9 years, aOR1.5 (1.01–2.3)), and receiving cotrimoxazole prophylaxis (10–14 years aOR 2.0 (1.2–3.6)). In conclusion, virological non-suppression is a factor of subsequent programme loss in both regions, and factors affecting the quality of care need to be addressed to achieve the third UNAIDS 90 in paediatric HIV.

## Introduction

South Africa has the largest paediatric human immunodeficiency virus (HIV) care and treatment programme in the world. In 2017, an estimated 280 000 children under 15 years of age were living with HIV in South Africa, of whom 58% were on antiretroviral therapy (ART) [1, 2]. Despite the rapid scale-up of access to ART in recent years, poor clinical outcomes remain a challenge. A previous study by Lilian *et al*. [3] showed that 20% of children who initiated ART between 2005 and 2014 in a rural South African area were lost to follow-up (LTFU) or had died, a minority, by 1 year on treatment.

The UNAIDS 90-90-90 targets include achieving viral suppression in 90% of children initiated on ART [4]. Unfortunately, there is a significant proportion of children that do not reach sustained viral suppression in response to first-line ART [5]. Previous studies found that psychosocial, clinical and treatment-related factors play a role in virological non-suppression in children [3, 6–8]. When looking at HIV-associated mortality, a study found that this was lower in urban areas compared with rural areas in both children and adults [9]. Poor viral suppression in childhood continues to undermine the goals of ART.

In South Africa, viral load (VL) testing to monitor response to ART is conducted at 6 and 12 months post-treatment initiation and then yearly if the VL is <50 copies/ml. VL testing is not performed at the time of ART initiation [10]. According to the Children with HIV Early antiretroviral (CHER) study, it is expected that >80% of children may achieve virological suppression following 6 months of ART and therefore, factors other than regimen effectiveness, are likely responsible for viral non-suppression [11]. There are challenges with obtaining specimens from children for VL testing. The skills of healthcare workers in primary healthcare settings may be limited with regards to paediatric phlebotomy, especially in younger children. This may lead to VL monitoring not taking place as insufficient or no specimens may be available for testing [12].

In order to ensure the delivery of high-quality care and treatment for children living with HIV, it is imperative to be able to identify children who are at higher risk of virological non-suppression. Factors associated with virological non-suppression may differ between age groups. Most studies combine children under 15 years as a homogenous group, however large psychosocial, developmental and clinical differences exist across this wide age range. In infants, the correct dosing of ART drugs as well as poor drug palatability may affect virological suppression, whereas, in adolescents, psychosocial issues and their effect on ART adherence appear to be the main factors affecting virological suppression [13].

The objective of this study was to assess the occurrence and associated factors of virological non-suppression in children on ART in South Africa, categorised by age, to identify priority groups and patient profiles for targeted interventions to improve the delivery of ART care.

## Methods

### Study population

**Fig. 1.**
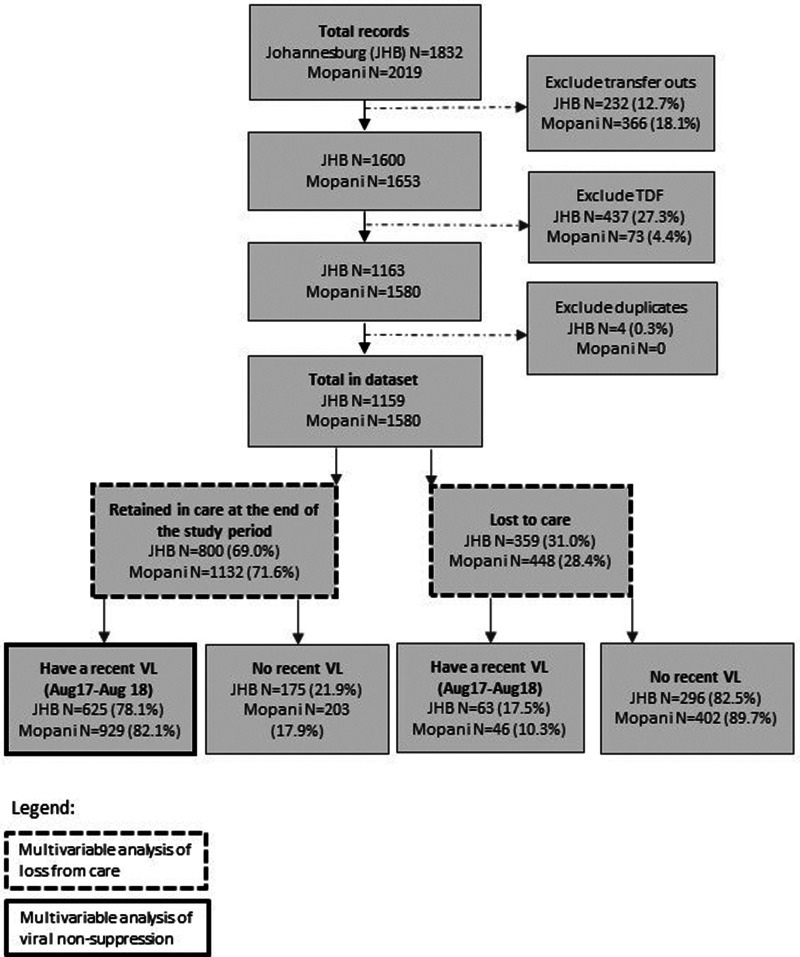
Flowchart of the study population.

The study was a retrospective cohort using TIER.Net (Three Interlinked Electronic Registers.Net) data. The South African National Department of Health implemented TIER.Net in 2010 to monitor the HIV programme by recording baseline and ongoing clinical care and treatment outcomes [14]. Data were routinely exported from TIER.Net for programme monitoring purposes. Anonymised TIER.Net data from two geographical areas were used for this analysis: rural Mopani District in Limpopo Province and four of seven sub-districts of urban Johannesburg Metropolitan District in Gauteng Province (sub-districts C, D, E and G). These districts and sub-districts were selected for convenience, as Anova Health Institute operated there as the designated United States Agency for International Development (USAID) district support partner during the study period. The data covered all public healthcare facilities offering paediatric ART services in the study areas. TIER.Net records were included for ART naïve children aged <15 years who were initiated on ART between July 2015 and August 2017, and who had complete data for three key variables (date of birth, date of ART initiation and date of last ART visit). The data was extracted in August 2018. Records of children who transferred out of the study area due to migration were excluded (*n* = 598). Tenofovir disoproxil fumarate (TDF) is only indicated for individuals aged ≥15 years according to South African guidelines at the time of the study [10]. Therefore, records were excluded if TDF was captured as part of the ART regimen (*n* = 510) to avoid misclassification of adults as children. In addition, duplicate records were excluded. The flowchart of the study population is presented in Figure 1. This analysis was approved by the University of the Witwatersrand's Human Research Ethics Committee (clearance number M140461) and the Johannesburg District Research Committee and Limpopo Provincial Health Research Committees of the Department of Health.

### Definitions and outcomes

The primary outcome was viral non-suppression *vs.* viral suppression among children retained on ART at the end of the follow-up period (August 2018). The 2015 South African ART guidelines define antiretroviral treatment failure (virological failure) as having two consecutive VL ≥ 1000 copies/ml [10], which is similar to the definition of the WHO. However, a single VL ≥ 1000 copies/ml would place a child at an increased risk of treatment failure and would need to be acted upon appropriately [10]. Therefore, an unsuppressed VL was defined as ≥1000 copies/ml for VL tests performed within a year of data extraction (between August 2017 and August 2018) in this study. This was also done to ensure that only recent test results were included in the analysis. Viral suppression was defined as a VL <1000 copies/ml.

Treatment status was the secondary outcome; defined as a loss to care *vs.* retained in care. Loss to care included children who were LTFU and those who died. In routine practice, the date for the next appointment is set at each ART clinic visit and we used this date in combination with the clinic's last TIER.Net update date to define treatment status. A child was defined as LTFU if the outcome was registered as such in TIER.Net or if the next appointment date was more than 90 days before the clinic's last data update, i.e. the child missed their appointment by more than 90 days. A child's death would be recorded in TIER.Net provided that this death was reported to the clinic. If a child died and this was not reported to the clinic, then the child may be classified as LTFU. A child was defined as retained in care when the next appointment date was after the clinic's last data update, or when the next appointment date was within 90 days before the clinic's last data update at the end of the study period. The time in months between the date of ART initiation and date of last ART visit was defined as time on ART.

### Statistical analysis

Viral suppression was assessed among children who were retained in care who had a recent VL on record. Independent factors of viral non-suppression were assessed using univariable and multivariable logistic regression analyses. The following variables were included: age at ART initiation, gender, age at the end of the study period, last cluster of differentiation 4 (CD4) cell count, time on ART (≤12 months, 13–24 months and >24 months), location (Mopani or Johannesburg), WHO clinical stage at ART initiation, baseline ART regimen, ART regimen at the most recent clinic visit and cotrimoxazole (CPT) prophylaxis at ART start. CD4 count in children older than 5 years was categorised in tertiles per age group. Variables with *P* < 0.10 were included in the multivariable model. Independent factors associated with loss to care status were also assessed with logistic regression using the same variables described for the analysis of viral non-suppression, but the total dataset was included in the analysis. All analyses were stratified by age group: less than 5 years, 5–9 years and 10–14 years, as psychosocial and clinical factors with respect to the provision of healthcare, differ for these age categories. SPSS version 24.0 (IBM, US) was used for analyses. A *P* value < 0.05 was considered significant.

## Results

### Characteristics of the study cohort

A total of 2739 ART-naïve children were initiated on ART in the study period, 42.3% (*n* = 1159) from the four sub-districts in Johannesburg and 57.7% (*n* = 1580) from Mopani District. The median age at ART initiation was 5 years (Interquartile range (IQR) 1–10 years), and 49.0% (*n* = 1342) of the children were male. At initiation, the median CD4 count among children older than 5 years of age was 729 cells/mm^3^ in Johannesburg (IQR 429–1321) and 727 cells/mm^3^ in Mopani (IQR 447–1258, *P* = 0.63). The median time on ART at the end of the study period was 20 months (IQR 12–27), which was comparable between the locations (*P* = 0.61). A total of 16.1% (*n* = 441) of children were on ART for less than 6 months, 9.1% (*n* = 249) between 6 months and 1 year, 41.6% (*n* = 1140) between 1 and 2 years, and 33.2% (*n* = 909) for more than 2 years.

### Loss to care

Of the 2739 children initiated on ART during the period, 70.5% (*n* = 1932) were retained in care, 25.9% (*n* = 711) were LTFU and 3.5% (*n* = 96) died. Early LTFU within 6 months of ART initiation was highest among children less than 5 years old (26.9%, *n* = 276), followed by children 5–9 years old (10.6%, *n* = 117) and children 10–14 years old (7.9%, *n* = 48, *P* < 0.001). The proportion of children who were reported to have died was higher in Mopani (7.1%, *n* = 87) *vs.* in Johannesburg (1.1%, *n* = 9, *P* < 0.001). Characteristics of children in care and children lost to care are presented in Table 1.

**Table 1.** Characteristics of children receiving ART, stratified by age categories, treatment outcome and viral suppression status

	Age < 5 years *N* = 1294				Age 5–9 years *N* = 665				Age 10–14 years *N* = 780				Total population *N* = 2739
Characteristics	In care *N* = 767			Loss to care^a^ *N* = 527	In care *N* = 547			Loss to care^a^ *N* = 118	In care *N* = 618			Loss to care^a^ *N* = 162	
% (*n*)/median (IQR)	Viral suppression *N* = 402	Viral non- suppression *N* = 178	No recent viral load^b^ *N* = 187		Viral suppression *N* = 327	Viral non- suppression *N* = 113	No recent viral load^b^ *N* = 107		Viral suppression *N* = 356	Viral non- suppression *N* = 178	No recent viral load^b^ *N* = 84		
ART initiation age	1 (0–3)	1 (0–3)	1 (0–2)	1 (0–2)	7 (6–8)	7 (6–8)	7 (6–8)	7 (6–8)	12 (11–13)	12 (11–13)	12 (11–13)	12 (11–13)	5 (1–10)
Current age	3 (2–5)	3 (2–4)	3 (2–4)	2 (1–3)	9 (8–11)	10 (8–10)	9 (8–10)	9 (7–10)	14 (12–15)	14 (13–15)	14 (13–15)	14 (12–15)	7 (3–12)
Sex % male	42.0 (169)	46.6 (83)	49.2 (92)	49.9 (263)	44.6 (146)	56.6 (64)	45.8 (49)	57.6 (68)	51.4 (183)	51.7 (92)	51.2 (43)	55.6 (90)	49.0 (1342)
Time on ART, months	25 (19–32)	22 (16–28)	24 (19–31)	4 (0–10)	22 (18–30)	24 (20–31)	23 (18–30)	7 (2–16)	22 (17–29)	23 (17–30)	22 (18–28)	9 (2–17)	20 (12–27)
WHO^c^ stage at initiation I	62.2 (250)	65.2 (116)	65.2 (122)	63.2 (333)	61.5 (201)	62.8 (71)	59.8 (64)	53.4 (63)	63.5 (226)	59.0 (105)	64.3 (54)	54.3 (88)	61.8 (1693)
WHO stage II	8.5 (34)	9.6 (17)	5.9 (11)	7.0 (37)	13.8 (45)	11.5 (13)	8.4 (9)	21.2 (25)	15.2 (54)	18.5 (33)	14.3 (12)	16.0 (26)	11.5 (316)
WHO stage III	9.2 (37)	8.4 (15)	8.0 (15)	8.0 (42)	9.2 (30)	11.5 (13)	11.2 (12)	10.2 (12)	7.0 (25)	11.2 (20)	11.9 (10)	16.0 (26)	9.4 (257)
WHO stage IV	1.7 (7)	1.7 (3)	2.1 (4)	2.7 (14)	2.4 (8)	2.7 (3)	1.9 (2)	3.4 (4)	2.5 (9)	1.1 (2)	0 (0)	3.7 (6)	2.3 (62)
WHO stage unknown	18.4 (74)	15.2 (27)	18.7 (35)	19.2 (101)	13.1 (43)	11.5 (13)	18.7 (20)	11.9 (14)	11.8 (42)	10.1 (18)	9.5 (8)	9.9 (16)	15.0 (411)
Location %Mopani	58.0 (233)	66.9 (119)	57.2 (107)	57.1 (301)	59.0 (193)	70.8 (80)	53.3 (57)	50.0 (59)	51.4 (183)	68.0 (121)	46.6 (39)	54.3 (88)	57.7 (1580)
Baseline treatment %1A3E	27.6 (111)	20.8 (37)	21.4 (40)	17.3 (91)	86.2 (282)	85.8 (97)	87.9 (94)	83.1 (98)	90.7 (323)	89.9 (160)	82.1 (69)	93.8 (152)	56.7 (1554)
Baseline treatment %1A3L	65.4 (263)	70.2 (125)	71.7 (134)	74.2 (391)	10.1 (33)	9.7 (11)	7.5 (8)	12.7 (15)	3.7 (13)	2.2 (4)	6.0 (5)	2.5 (4)	36.7 (1006)
Other treatment	7.0 (28)	9.0 (16)	7.0 (13)	8.5 (45)	3.7 (12)	4.4 (5)	4.7 (5)	4.2 (5)	5.6 (20)	7.9 (14)	11.9 (10)	3.7 (6)	6.5 (179)
CPT prophylaxis at ART start % yes	13.2 (53)	15.7 (28)	8.0 (15)	13.5 (71)	8.6 (28)	15.0 (17)	9.3 (10)	11.9 (14)	8.7 (31)	17.4 (31)	17.9 (15)	19.8 (32)	12.6 (345)
CD4 cells/mm^^c^^	na	na	Na	na	528 (264–869)	410 (238–658)	557 (193–817)	518 (313–884)	445 (227–750)	321 (145–546)	362 (137–509)	302 (14 5–527)	432 (2010–727)

WHO, World Health organization; A3E, abacavir, lamivudine and efavirenz; A3L, abacavir, lamivudine and lopinavir/ritonavir; CPT, cotrimoxazole prophylaxis.

a Loss to care included children LTFU and those who died.

b This included viral loads done more than one year ago.

c CD4 counts were available for 419 children from 5–9 years and 513 children from 10–14 years. Viral loads of children who were loss to care were available for 201, 71 and 93 children per age group respectively.

### Loss to care and the association with virological non-suppression

For children lost to care, only 45.2% (365/807) had any VL available at any given time point; 46.8% (333/711) for children LTFU and 33.3% (32/96) for children who died. Non-suppressed children (30.3%, 229/755) were more often lost to care compared with suppressed children (9.7%, 136/1399, *P* < 0.001). Virological non-suppression was highest in children who died (100%, *n* = 6), followed by children LTFU (51.5%, *n* = 53). In multivariable analyses, shorter time on ART (≤12 months) and not having a recorded VL or VL >1000 copies/ml were independently associated with higher loss to care in all age groups (Table 2). In children under 5 years of age, receiving treatment in Johannesburg was also independently associated with loss to care.

**Table 2.** Factors independently associated with loss to care identified by multivariable logistic regression analysis, stratified by age groups

	Loss to care^a^ 29.5% (807/2739)					
	<5 years 40.7% (527/1294)		5–9 years 17.7% (118/665)		10–14 years 20.8% (162/780)	
	Loss to care % (*n*)	aOR (95% CI)	Loss to care % (*n*)	aOR (95% CI)	Loss to care % (*n*)	aOR (95% CI)
Age at ART start						
0 years	41.0 (188)	1	18.9 (20)	5 years 1	10 years 15.1 (25)	ns
1 year	35.1 (101)	0.8 (0.5–1.3)	19.3 (21)	6 years 1.2 (0.5–3.1)	11 years 12.5 (22)	–
2 years	33.3 (59)	0.6 (0.3–1.1)	5.2 (7)	7 years 0.5 (0.2–1.3)	12 years 15.2 (22)	–
3 years	22.6 (30)	0.4 (0.2–0.8)	14.6 (21)	8 years 0.9 (0.4–2.2)	13 years 18.0 (22)	–
4 years	24.8 (29)	0.8 (0.4–1.7)	14.1 (20)	9 years 0.6 (0.2–1.5)	14 years 13.8 (16)	–
Sex						
Male	38.6 (216)	1	16.5 (51)	1	15.4 (58)	ns
Female	31.1 (191)	1.2 (0.8–1.7)	11.7 (38)	0.6 (0.3–1.1)	14.0 (49)	–
Time on ART						
≤12 months	90.7 (342)	91.5 (51.8–161.5)	74.7 (68)	66.9 (28.1–155.3)	72.9 (99)	60.4 (27.2–134.3)
13–24 months	12.2 (50)	2.1 (1.3–3.4)	5.5 (17)	1.7 (0.8–3.7)	8.6 (32)	2.8 (1.5–5.5)
>24 months	3.9 (15)	1	1.7 (4)	1	1.9 (5)	1
WHO stage						
WHO stage at initiation I	34.5 (257)	ns	12.7 (49)	ns	14.1 (63)	1
WHO stage II	31.9 (29)	–	23.0 (20)	–	16.8 (20)	1.7 (0.9–3.4)
WHO stage III	30.2 (29)	–	12.7 (8)	–	14.1 (9)	1.6 (0.7–3.5)
WHO stage IV	39.1 (9)	–	13.3 (2)	–	31.3 (5)	2.3 (0.5–11.6)
WHO stage unknown	37.9 (83)	–	11.6 (10)	–	12.8 (10)	1.5 (0.7–3.5)
CPT at ART start						
		ns		ns		
No	41.9 (386)	–	18.5 (92)	–	19.3 (108)	1
Yes	42.5 (71)	–	20.3 (14)	–	29.4 (32)	2.1 (1.1–4.1)
Not available	34.0 (70)	–	12.2 (12)	–	19.8 (22)	0.9 (0.4–1.8)
Location						
Johannesburg	57.7 (308)	2.0 (1.4–3.0)	14.9 (38)	1.0 (0.6–1.9)	15.4 (50)	1.6 (0.9–2.6)
Mopani	42.9 (226)	1	13.4 (51)	1	14.3 (57)	1
Recent viral load copies/ml						
<400	8.3 (39)	1	5.3 (20)	1	3.3 (12)	1
400–1000	14.3 (8)	2.2 (0.9–5.1)	3.1 (1)	0.9 (0.2–4.3)	7.0 (3)	0.9 (0.3–3.3)
>1000	31.4 (96)	2.3 (1.4–3.7)	16.1 (24)	3.8 (1.9–7.5)	18.4 (43)	5.6 (3.1–10.2)
Not recorded	76.7 (264)	5.4 (3.2–8.9)	57.1 (44)	4.6 (2.0–10.6)	62.0 (49)	11.5 (5.5–24.0)

aOR, adjusted odds ratio; ART, antiretroviral therapy; WHO, World Health Organization; CPT, cotrimoxazole prophylaxis; CI, confidence interval; na, not applicable; ns, non-significant in univariable analyses and therefore not included in the multivariable model.

a Loss to care included children LTFU and children who died.

### Virological non-suppression

The virological non-suppression analysis only included children retained in care who had a recent VL result from testing performed within a year of the end of the study period. Of the 1932 children retained in care, 80.4% (*n* = 1554) had a recent VL result: 78.1% (625/800) from Johannesburg and 82.1% (929/1132) from Mopani. Non-suppression based on the most recent VL test was observed in 30.2% (469/1554) of all children in care; 23.8% (149/625) Johannesburg and 34.4% (320/929) in Mopani (*P* < 0.001). Viral non-suppression differed across the age categories: 30.7% (*n* = 178) in children under 5 years, 25.7% (*n* = 113) in children from 5 to 9 years and 33.3% (*n* = 178) in children from 10 to 15 years (*P* = 0.03).

### Independent factors associated with virological non-suppression per age category

In children under 5 years old, shorter time on ART (<12 months and 13–24 months compared to >24 months) and receiving treatment in Mopani District were independently associated with virological non-suppression (Table 3). In children aged 5–9 years, being male and receiving treatment in Mopani were independently associated with virological non-suppression. In children aged 10–14 years, receiving treatment in Mopani, receiving a first-line regimen other than abacavir, lamivudine and efavirenz (1A3E) or abacavir, lamivudine and lopinavir/ritonavir (1A3L) at baseline, and having a lower or not recorded CD4 cell count were independently associated with virological non-suppression.

**Table 3.** Factors independently associated with virological non-suppression identified by multivariable logistic regression analysis, stratified by age groups

	Virological non-suppression 30.2% (469/1554)					
	<5 years 30.7% (178/580)		5–9 years 25.7% (113/440)		10–14 years 33.3 (178/534)	
	% (*n*) non-suppression	aOR (95% CI)	% (*n*) non-suppression	aOR (95% CI)	% (*n*) non-suppression	aOR (95% CI)
Sex		ns				ns
Male	32.9 (83)		30.5 (64)	1.5 (1.0–2.3)	33.5 (92)	
Female	29.0 (95)		21.3 (49)	1	33.2 (86)	
Time on ART				ns		ns
≤12 months	50.0 (14)	3.7 (1.6–8.2)	0.0 (0)^a^		20.8 (5)	
13–24 months	35.5 (97)	1.8 (1.3–2.7)	26.1 (61)		32.6 (95)	
>24 months	24.0 (67)	1	27.7 (52)		35.8 (78)	
Location						
Johannesburg	25.9 (59)	1	19.8 (33)	1	24.8 (57)	1
Mopani	33.8 (119)	1.7 (1.1–2.4)	29.3 (80)	1.8 (1.1–3.0)	39.8 (121)	1.9 (1.2–2.8)
CPT at ART start		ns		ns		
No	30.7 (122)		24.8 (82)		30.9 (122)	1
Yes	34.6 (28)		37.8 (17)		50.0 (31)	2.0 (1.2–3.6)
Not available	27.5 (28)		21.9 (14)		32.5 (25)	1.1 (0.7–2.0)
Last CD4 cells/mm^3^^b^		ns				
Lowest tertile	29.7 (41)		34.7 (33)	2.1 (1.1–4.1)	38.8 (71)	2.1 (1.2–3.8)
Middle tertile	24.0 (18)		26.3 (25)	1.5 (0.7–2.9)	33.3 (39)	1.7 (1.0–3.1)
Highest tertile	26.0 (19)		18.9 (18)	1	21.7 (25)	1
Not recorded	34.0 (100)		23.9 (37)	1.1 (0.6–2.1)	37.1 (69)	1.7 (1.0–2.9)

aOR, adjusted odds ratio; ART, antiretroviral therapy; CPT, cotrimoxazole prophylaxis; CI, confidence interval; na, not assessed; ns, non-significant in univariable analyses and therefore not included in the multivariable model.

a This category was excluded in univariable analysis as there were no cases of non-suppression.

b Categories were based on tertiles per age group: 5–9 years (≤365, 366–705, ≥706); 10–14 years (≤272, 273–555, ≥556).

## Discussion

This study evaluated virological non-suppression and ART programme retention of HIV-infected children in two settings in South Africa. High levels of retention and virological suppression are imperative for good programme outcomes, however, we show that a substantial number of children fail to virally suppress after ART initiation and are lost to the programme within a relatively short period after ART initiation. Of concern, virological non-suppression is a factor of subsequent programme loss, and factors affecting the quality of care need to be addressed to achieve the third UNAIDS 90 in paediatric HIV.

Almost one-third of the children enrolled in the HIV programme were lost to care at the end of the 2 year study period. The proportion of children that were LTFU (26%) was somewhat higher compared with findings from a systematic review among other African countries (median 17%, range 2.6–57.4%) [9]. The most common definition for LTFU in this review was ‘not seen for at least 3 months’, which is comparable to our follow-up time (>90 days after previous visit). Independent factors for loss to care for all age groups were receiving ART for less than a year and having a recent high VL. Therefore, interventions that focus on children who recently initiated ART could be useful to prevent early loss to care. This is also important for children with high VLs, who are at increased risk of developing immune suppression as well as becoming ill and dying. HIV-infected children and adolescents in care need dynamic, innovative and multidisciplinary approaches to improve retention [9, 15, 16].

Among children who had a recent VL, almost one-third of children experienced virological non-suppression in their first years on ART. This corresponds with recent studies (2017–2019) from low- and middle-income countries (LMICs) (28–38%) [5, 17–22]. However, two systematic reviews (both 20%) [22, 23] and other studies have found lower proportions of virological non-suppression in children, including 12% in Ethiopia [6], 18% in South Africa [15] and 20% in Thailand [24].

The South African HIV management guidelines call for VL monitoring at 6, 12 and 24 months (if the previous VL result was <50 c/ml) following ART initiation [10], therefore all children should have a VL performed at least yearly. VL monitoring is a priority for the early identification of virological non-suppression and regimen evaluation [25]. Despite the availability and implementation of VL testing, regular VL monitoring remains challenging in children [17]. This may be improved through interventions such as the VL champion programme which significantly enhanced monitoring in a South African setting [26].

Receiving treatment in a healthcare clinic in the rural district was independently associated with virological non-suppression across all age groups compared to urban facilities. A possible explanation is the lower skills level and fewer healthcare professionals per population size in rural areas [3]. This may result in poorer quality of care, including adherence counselling and other appropriate responses to unsuppressed VLs (e.g. regimen switching). In addition, social determinants of health could play a role such as poorer access to transport and food, which could impact treatment adherence and health-seeking behaviour.

The strengths of our study are the use of the TIER.Net database which enabled a multi-site programme analysis of the largest HIV programme worldwide, enhancing insights into the performance of the paediatric HIV programme in a rural and urban setting. Moreover, a substantial number of children (*n* = 2739) were included over a 2-year period, enabling regression analyses and stratification for age groups.

The study also has limitations. It is possible that not all clinical data were correctly captured and that some data were not captured at all, resulting in missing data; this is a general limitation of using routine data. This explains the relatively larger proportion of Mopani children excluded for meeting the study criteria as essential variables were not captured. Almost one-third of children who were lost to care did not have any recorded VL results. It is likely that these children were not suppressed because of early dropout or the children did not attend for follow-up. It is unclear how this would affect the results of this study. Also, the recording of death as a treatment outcome relies on deaths being reported to the health facility and captured, therefore more of those lost to care could have died. Analyses were stratified by age groups based on treatment regimens and authors' clinical expertise, and it is possible that results could vary when choosing other cut-offs. Finally, this study bases virological non-suppression on a single recent VL test and this could lead to an overestimation of virological failure, which is defined by the WHO as two consecutive VL results ≥1000 copies/ml. However, the proportion of children experiencing non-suppression was somewhat comparable to other studies in LMICs (see the previous paragraph).

In conclusion, this study provides a valuable evaluation of clinical care addressing the third UNAIDS 90-90-90 target [4], viral suppression. One in three children was lost to care and one in four experienced virological non-suppression indicating there is a strong need to improve the quality of care for children starting ART. In particular, children with an unsuppressed VL, children seeking healthcare in rural settings and children with advanced stages of disease need interventions to improve retention in care and viral suppression. Adequate monitoring of paediatric VLs is imperative to improve viral suppression and long-term outcomes in South African children living with HIV.

## Data Availability

Any data sharing requests should be directed to the Anova Health Institute (dunlop@anovahealth.co.za). Restrictions apply to the availability of these data may be applicable.
